# Participants’ Perceptions of Essential Coaching for Every Mother—a Canadian Text Message–Based Postpartum Program: Process Evaluation of a Randomized Controlled Trial

**DOI:** 10.2196/36821

**Published:** 2022-05-13

**Authors:** Justine Dol, Megan Aston, Douglas McMillan, Gail Tomblin Murphy, Marsha Campbell-Yeo

**Affiliations:** 1 Faculty of Health Dalhousie University Halifax, NS Canada; 2 St. Michael's Hospital Toronto, ON Canada; 3 School of Nursing Faculty of Health Dalhousie University Halifax, NS Canada; 4 Department of Pediatrics Dalhousie University Halifax, NS Canada; 5 Nova Scotia Health Halifax, NS Canada

**Keywords:** mHealth, text messaging, postpartum, process evaluation, mobile health, SMS, text message, digital health, randomized control trial, postnatal

## Abstract

**Background:**

“Essential Coaching for Every Mother” is a Canadian text message–based program that sends daily messages to mothers for 6 weeks after they give birth. There is a need to explore the program’s effectiveness in terms of the participants’ experience to guide refinement and modification.

**Objective:**

This study aimed to describe the process evaluation of the Essential Coaching for Every Mother randomized controlled trial through an evaluation of the research implementation extent and quality.

**Methods:**

Participants were recruited from Nova Scotia, Canada, between January 5 and August 1, 2021. Enrolled participants were randomized into the intervention or control group. Participants randomized to the intervention group received standard care along with the Essential Coaching for Every Mother program’s text messages related to newborn and maternal care for the first 6 weeks after giving birth, while the control group received standard care. Usage data were collected from the SMS text message program used, and participants completed web-based questionnaires at 6 weeks after birth. ﻿Quantitative data and qualitative responses to open-ended questions were used to triangulate findings. Quantitative data were summarized using means, SDs, and percentages, as appropriate, while qualitative data were analyzed using thematic analysis.

**Results:**

Of the 295 unique initial contacts, 150 mothers were eligible and completed the baseline survey to be enrolled in the study (intervention, n=78; control, n=72). Of those randomized into the intervention group, 75 (96%) completed the 6-week follow-up survey to provide feedback on the program. In total, 48 (62%) intervention participants received all messages as designed in the Essential Coaching for Every Mother program, with participants who enrolled late missing on average 4.7 (range 1-12) messages. Intervention participants reported an 89% satisfaction rate with the program, and 100% of participants would recommend the program to other new mothers. Participants liked how the program made them feel, the format, appropriate timing of messages, and content while disliking the frequency of messages and gaps in content. Participants also provided suggestions for future improvement.

**Conclusions:**

Our process evaluation has provided a comprehensive understanding of interest in the program as well as identified preference for program components. The findings of this study will be used to update future iterations of the Essential Coaching for Every Mother program.

**Trial Registration:**

ClincalTrials.gov NCT04730570; https://clinicaltrials.gov/ct2/show/NCT04730570

## Introduction

Mothers undergo significant changes during the postpartum period, physically as well as emotionally and relationally, as they adjust to their new mothering role [1,2]. Rates of postpartum anxiety and depression are high, with approximately 17% of mothers reporting postpartum depression symptoms [3] and 15% reporting postpartum anxiety symptoms [4], suggesting the postpartum period is a particularly vulnerable time period for mothers’ mental health. During this critical period, mothers learn the skills of motherhood, which can influence how mothers see themselves in relation to their new infant [5] and influence their perception of maternal self-efficacy [6]. During the postpartum period, mothers can often feel undersupported, unsure about their new role as a mother and struggle to find reliable information about caring for their infant [7,8]. Mothers who do not have enough support nor information to help in the transition to motherhood may experience challenges in their psychosocial adjustment and conceptualization of their self-efficacy as a mother [8,9].

To assist with the transition after birth, interventions that target postpartum adjustment and health outcomes are important, with mobile health (mHealth) being one innovative strategy that can be used to provide postpartum education directly to mothers. mHealth is defined as the use of mobile devices, such as mobile phones or smartphones, to transmit various health content and services [10] and can be used across multiple health outcomes and conditions. In the current context, mHealth interventions can be used to complement existing postpartum care, enhancing maternal self-efficacy and feelings of social support through the provision of standardized, time-appropriate, and evidence-based information.

Globally, mHealth interventions have been used to target the perinatal period, with varied impact on maternal psychosocial and newborn outcomes [11-14]. One systematic review on mHealth interventions in high-income countries (n=21) found significant variation in approach and intervention, with positive impacts on postpartum depression for mHealth interventions targeting this outcome [11]. While there are some mobile health interventions targeting the antenatal period [15], there are none currently available that have been evaluated that target the postpartum period in Canada [11]. In the abovementioned systematic review [11], only one study was conducted in Canada, which made phone calls to women with postpartum depression [16]. In a recent study in the Maritime provinces, which includes Nova Scotia where the current study was conducted, 61% of women reported low parenting self-efficacy, 31% had high postpartum anxiety, and 52% had depressive symptoms [17], suggesting that there is a need for a targeted intervention in this area.

While evidence suggests that mHealth innovations can improve health outcomes in the postpartum period [11], there is a need to further explore a program’s effectiveness, through the participant’s experience, to guide refinement and modification. While the use of a randomized controlled trial (RCT) design is considered to be a gold standard to examine the effect of an intervention, a limitation of this design can be that the findings fail to explain the underlying process and context associated with the implementation of the intervention [18]. There is growing recognition that it is not enough to know “if a health intervention is effective; it is also necessary to understand why the intervention works, how, for whom and in which contexts” [19]. The goal of process evaluation in RCTs is to identify the factors that influence success or failure during implementation by taking into account the complexity of health behavior interventions to identify contextual factors associated with variation in outcomes [20-22].

As part of a hybrid type 1 effectiveness implementation RCT [23], this study aims to describe the process evaluation component of the Essential Coaching for Every Mother RCT; in particular, (1) the research implementation extent (eg, number of participants recruited, timing of recruitment) and (2) implementation quality measured through the likes and dislikes of the program from the perspective of participants and suggestions for further improvement.

## Methods

### Intervention

To capitalize on the potential of mHealth to support postpartum mothers, we developed the Essential Coaching for Every Mother program, which includes 53 SMS text messages sent over the first 6 weeks after birth, which are related to newborn care and maternal mental health [24]. The program is designed to send 2 SMS text messages per day in the first 2 weeks and a daily message for weeks 3 through 6. Messages are sent automatically on the basis of the newborn’s date of birth. In both the pilot feasibility study [25] and the RCT (currently under review), the Essential Coaching for Every Mother program was found to improve maternal self-efficacy and decreased postpartum anxiety.

### Participants

Participants were recruited from Nova Scotia, Canada, between January 5 and August 1, 2021, for an RCT on the Essential Coaching for Every Mother program. Participants were eligible if they (1) were between 37+0 weeks pregnant and 10 days postpartum, (2) had daily access to a mobile phone with texting capabilities, (3) were over 18 years of age, (4) lived and gave birth in Nova Scotia, and (5) spoke and could read English. Participants were excluded if (1) their newborn died or was expected to die prior to leaving the hospital, (2) they did not have access to a mobile phone (either personal or shared), (3) they were unwilling to receive SMS text messages, (4) declined or withdrew by not completing the baseline survey, or (5) previously participated in the development or feasibility phase of this project. Additional details about the study are reported elsewhere [23].

Of note, the use of the term “mothers” is in its broadest sense to refer to any birthing individual, recognizing that not all birthing individuals identify as mothers and not all mothers identify as women [26,27]. All individuals who physically gave birth were recruited to participate in this study, regardless of gender identity.

### Ethics Approval and Study Registration

This study was approved by the IWK Health Research Ethics Board (1024984) and Nova Scotia Health Research Ethics Board (1026534) and is registered with the ClinicalTrials.gov Protocol Registration System (NCT04730570).

### Study Procedures

This was a 2-group, stratified, parallel-arm RCT following a predefined protocol [23]. Recruitment occurred remotely using both web-based platforms and posters for study promotion. Participants could initiate contact during pregnancy (considered antenatal recruitment) or after the birth of their infant (considered postpartum recruitment). All contact with participants occurred through a predesigned SMS text message flow system in TextIt [28], with sending facilitated through Twilio [29]. TextIt is a web-based interface platform where messages can be preprogrammed in a time-based flow, which is compatible with Twilio, a gateway service that offers virtual phone numbers that send and receive messages on behalf of TextIt [28,29]. A researcher only engaged with a participant during recruitment if they asked a question that was not understood by the predesigned program. No interaction with participants occurred while they were in the program. All eligibility criteria were self-reported by potential participants as they passed through screening questions via SMS text messages prior to enrollment in the study. Participants also self-reported the date that they gave birth to their infant, which was prompted at set times if a participant was recruited antenatally (eg, weekly between 39 and 42 weeks) or during the recruitment flow if recruited postpartum.

### Recruitment

**Figure 1:**
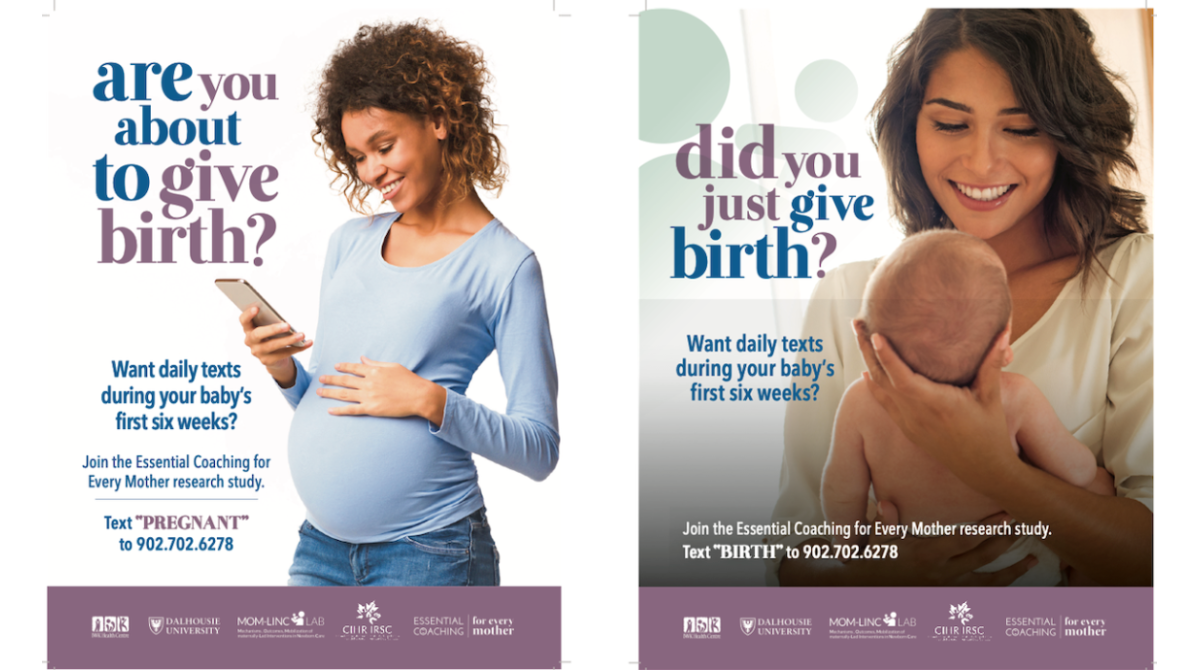
Recruitment posters for antenatal and postpartum recruitment for hospitals.

**Figure 2:**
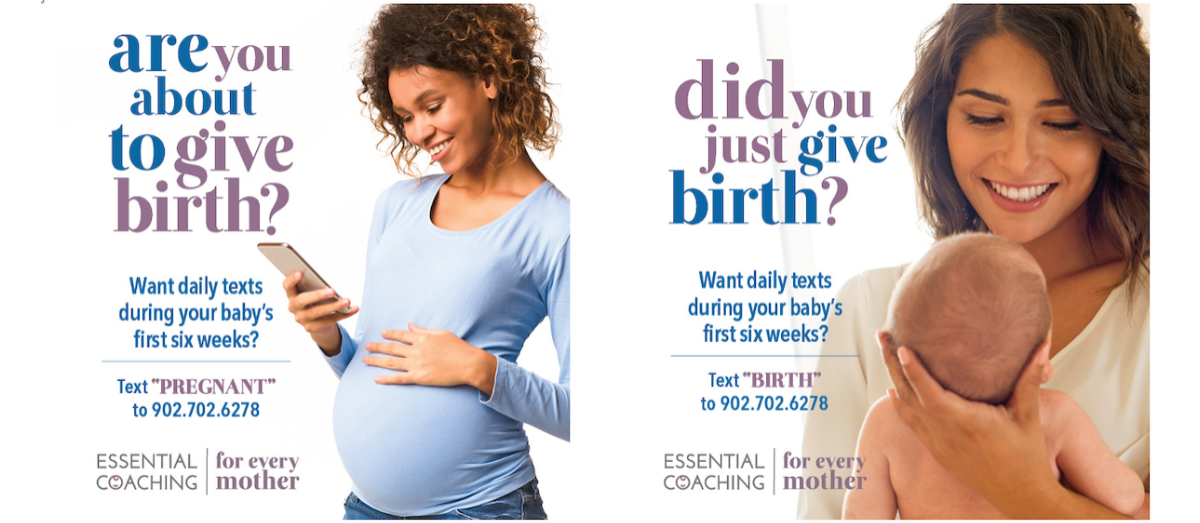
Social media images for antenatal and postpartum recruitment.

Recruitment occurred through study posters at local hospitals, paid and unpaid social media advertisements (eg, Facebook, Instagram, and Twitter), sharing through family resource centers, and relevant organizations (eg, local public health units and baby stores). Figure 1 illustrates the antenatal and postpartum recruitment posters that were used in hospitals and clinics, with Figure 2 illustrating the modified version that was used with social media advertisements and posts. For the paid Facebook advertisements, approximately CAD $780 was spent over the campaign. These paid advertisements targeted women aged 18-45 living in Nova Scotia.

After recruitment, participants provided consent and were randomized into the intervention group (Essential Coaching for Every Mother program) or standard care. Participants in both the intervention and control group were requested to complete the consent form, baseline survey (enrollment after birth), 6-week follow-up survey, and 6-month follow-up survey. Additional details about study procedures and the RCT are available elsewhere [23].

### Outcome Measures

To measure the extent of implementation of the Essential Coaching for Every Mother program, output data available through the Twilio and TextIt platforms [28,29] were collected per participant, including the enrollment rate (ie, percentage contacted compared to percentage enrolled, and number of withdraws), enrollment timing (ie, postpartum or antenatal and days post partum), and numbers of messages received based on enrollment timing (per participant). These data were collected throughout the trial.

To measure implementation quality, in the 6-week survey, mothers in the intervention group were asked about user experience, perspectives on the frequency and timing of messages, and what did they like and not like about the Essential Coaching for Every Mother program. Using these two approaches together seeks not only to obtain data on the implementation extent but also on the quality through open-ended questions where mothers provided feedback on their experience with the Essential Coaching for Every Mother program in practice.

### Data Analysis

Summative data were reported through means, SDs, and percentages as appropriate. Open-ended questions from the survey were analyzed using thematic analysis [30] led by the first author.

## Results

### Implementation Extent

During the recruitment period, 295 initial contact messages were sent to the study phone number. Of them, 43 declined to participate (ie, did not complete the screening process), 37 contacted the study number after recruitment was concluded, and 44 did not meet the eligibility criteria.

**Figure 3:**
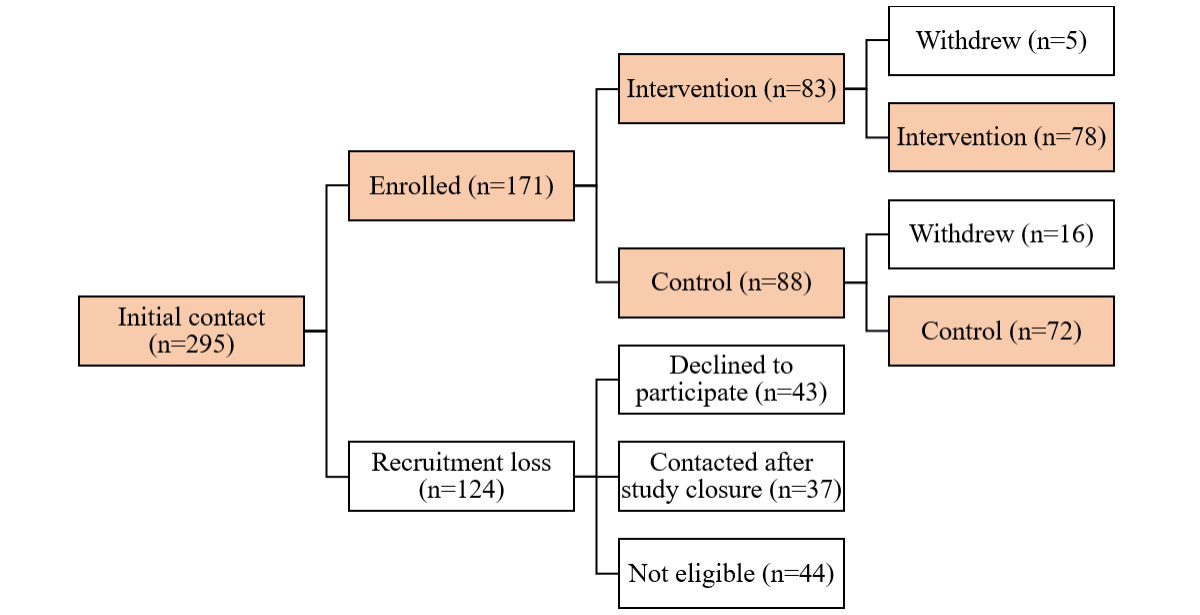
Enrollment flow for participants who completed baseline assessment.

Of the 171 participants randomized, 83 were randomized to the intervention arm and 88 to the control arm. Two participants texted “STOP” to withdraw from the program and 19 did not complete the baseline survey and were thus considered to have withdrawn from the trial after randomization. Of them, 16 were from the control arm and 5 were from the intervention arm. Figure 3 shows the enrollment flow diagram. In total, 150 participants (50.8%) who contacted the study number were randomized and completed the baseline survey.

For these 150 participants, the mean age of the newborn at enrollment was 2.1 (SD 2.6) days and mothers had a mean age of 31.4 (SD 4.5) years. Most participants identified as White (n=132, 88%) with 18 participants (12%) identifying as landed immigrants. Most participants contacted the study number (n=100) antenatally, with 50 having contacted the study number during the postpartum period. Most enrolled participants found out about the study through posters at the IWK Health Centre in the postpartum rooms (n=64, 42.7%) or perinatal clinics (n=7, 4.7%), followed by Facebook, either posts in groups (n=38, 25.3%), paid advertisements (n=17, 11.3%), or on Marketplace (n=2, 1.3%). Other approaches included word of mouth from family or friends (n=11, 7.3%), family resource centers (n=4, 2.6%), Instagram (n=3, 2.0%), or Kijiji (n=1, 0.1%). Three participants (2.0%) did not specify where they heard about the study.

Of the 78 participants who were randomized to the Essential Coaching for Every Mother program, 48 (61.5%) received full SMS text messages. Of them, 39 (81.3%) were antenatally recruited and 9 (18.7%) were recruited postnatally. The 30 participants who enrolled after the program was designed to start, missed on average 4.7 (SD 3.9, range 1-12) messages. This corresponds to missing on average 2.5 days of messages, on average starting the messages on day 5 post partum. Of them, 18 (60.0%) were antenatally recruited and 12 (40.0%) were recruited postnatally.

### Implementation Quality

Among the participants who received the Essential Coaching for Every Mother program and completed the 6-week follow-up survey (n=75), 65 (86.7%) felt that the number of messages was just right, while 6 (8.0%) felt that there were too few messages, and 4 (5.3%) felt that there were too many messages. Overall, participants reported an 89.2% satisfaction rate with the program, and 100% of participants would recommend the program to other new mothers.

Most participants (n=65, 86.7%) felt that the messages reflected all the information needs they had related to their own postpartum experience. Areas of additional informational need related to the maternal postpartum experience were sleep, physical recovery after childbirth (both vaginal and caesarian), breast milk pumping, return of menses, and where to go for postpartum support. Similarly, most participants (n=66, 88.0%) felt the messages reflected all the information needs that they had related to caring for their newborn. Additional information areas requested were primarily related to breastfeeding or pumping as well as newborn sleep, secure attachment, soothing, infant cardiopulmonary resuscitation (CPR), and when to seek medical help.

When asked what participants liked most about the Essential Coaching for Every Mother program, responses fell into four categories: general praise, how the messages made them feel, the format, and the appropriateness of message timing and content. In terms of general praise, participants provided nonspecific appreciation for the program, stating that the information provided was “amazing” and “informative.” Participants also commented on how the program made them feel, in that they felt reassurance and support from the program, with some even commenting that it felt like a daily check-in. One mother said the following: “As a first-time mom, it was reassuring to see some of the information and to get resources,” while another said, “I liked that it made me feel connected to something.”

Participants also commented that they appreciated the format of the program, particularly liking that the information came via SMS text message and at standard times. One participant explained the following:

Initially when I was receiving two a day, I was excited for 10am and for 5pm... I was sad when I realized the 5pm ones stopped! I enjoyed receiving the texts because it was something to look forward to.

The timing and content of messages was another area that participants really liked about the program. Participants commented that the messages were timely and contained information relevant to their stage of their newborn:

Sometimes I would be thinking about something and receive a text about that exact subject matter! It was almost like they read my mind.

Participants also provided praise on the general content of the information and that links were provided for additional sources of support. One participant appreciated that the program was “Concise, Canadian, [with] good information conveyed.” Another participant said, “I also liked the links to more information that were provided, like the video about purple crying and pelvic floor therapy.”

Among the 75 participants who received the program, 41 (55.7%) provided feedback about what they did not like about the program. The primary areas that participants did not like fell into two main categories: frequency of messages and gaps in content. In relation to the frequency of messages, some participants felt that there were too many messages, some were repetitive, and that the messages were sent in batches of 3, rather than as 1 long message. One participant said, “It came in multiple messages. I would have preferred if it came in one big message, so I didn't get as many notifications.” On the other hand, a few participants desired more messages: “I would have liked more messages as it was very comforting.”

In terms of content gaps, participants commented on some missing content, such as additional information on breastfeeding, newborn sleep, or on postpartum health for the mothers. Some participants commented that not all information was relevant to them, or they would have liked additional information or support on some topics. One mother said, “some texts didn't provide enough detail or where to find additional information,” while another explained that she wanted to know “If there was a way to seek further support besides just a public health nurse.” A few participants felt that information regarding the COVID-19 pandemic was too much. Furthermore, others wished that the program was more interactive: “I wish I could text back and forth and ask questions.”

Finally, participants were asked to provide areas where the Essential Coaching for Every Mother program could be improved, which were categorized into 4 main areas. The first related to extending the program, both beyond 6 weeks and to other family members and care providers. One participant said, “I would have liked to continue to get messages further than 6 weeks as they were very helpful.” The second area for improvement was to provide the option to tailor the content, whether to reduce or increase the frequency of messages, or to provide more, or less, content about a particular topic. Some participants preferred to receive more messages—“maybe texts twice a day to get more information”—while others preferred fewer messages—“1 message a day.” One mother suggested, “Maybe tailored to mothers pre-existing knowledge or some specifics for second time moms.” The third area for improvement was to provide additional links or areas for external support for web-based or local resources. Participants wanted more information about accessing support—“including more links for follow up information”—and more information in the texts themselves—“Maybe more links to pdf files with basic information. Not everything can be conveyed via text but sometimes more info is required.” The final area for improvement was to provide an interactive component, where participants could respond to the SMS text messages or engage with other mothers. One participant summarized, “The information was all amazing but being able to interact would be great as well. If there's any questions to be able to respond to the texts or speak to other mothers.”

## Discussion

### Principal Findings

This paper presents the process evaluation of the Essential Coaching for Every Mother RCT conducted in Nova Scotia, Canada. Almost two-thirds of participants received the full program as it was designed, with participants who enrolled later missing, on average, approximately 2.5 days of messages. Participants who received the program were generally satisfied and all would recommend it to other new mothers. Likes and dislikes related to the program were identified, along with areas for future improvement.

### Comparison With Other Studies

Similar to the feasibility study [25], there was significant interest in the Essential Coaching for Every Mother program. Recruitment occurred quickly (8 months) through the use of completely remote, passive strategies. It appears that posters in the hospital were the most successful strategy, followed by posts and advertisement through Facebook. Several reviews have identified that the use of Facebook to recruit for health research can prove fruitful, especially for hard-to-reach populations [31-33]. This is particularly relevant given that this study’s recruitment occurred during the COVID-19 pandemic, which significantly limited the ability to carry out in-person recruitment in hospital as was originally planned. However, a limitation of recruitment via social media is the lack of generalizability and overrepresentation of White, middle-higher–income participants [31-33]. Therefore, the supplementation of remote recruitment through posters in the postpartum unit of local hospitals allowed for the promotion of the study without requiring physical interaction with potential participants by research staff.

In the feasibility study examining the program, less than half of the participants received the full messages [25], compared to almost two-thirds in this study. This could be partially explained as this study specifically targeted recruitment during the antenatal period on the basis of lessons learned during the feasibility study, which seems to have worked in terms of ensuring that more participants received the full design of messages. What is interesting is that even though two-thirds of participants were recruited antenatally, almost half of participants indicated that they heard about the study via the study poster in the postpartum room. This suggests that participants actually heard about the study earlier and enrolled while they were still pregnant but were reminded about the study through the poster in their room after they gave birth, triggering their engagement with the program shortly after birth. The question about where participants heard about the study occurred in the baseline survey after they gave birth, making the study poster the most recent reminder about the study, potentially influencing their response. Nevertheless, 38.5% of participants enrolled after the program was designed to start (ie, the evening of the second day after birth), starting on average on day 5 post partum, suggesting that perhaps a reconsideration for the design start time may be needed. While the missed messages did not impact outcomes in the feasibility study, and the study team did see improvement in enrollment timing between the feasibility and RCT, this is a large number of participants who did not receive the full program as designed.

Responses from the 6-week follow-up survey were positive and demonstrate that the Essential Coaching for Every Mother program was valuable to mothers. Overall, 89% of participants felt that the number of messages was just right and most of the content addressed what they needed to know related to caring for themselves and their newborn after birth. Identifying the appropriate length and frequency of an SMS text message program is a challenge as there is no standardized evidence of what would be a perfect length for SMS text message programs in relation to the length of the program and frequency of messages. Another Canadian SMS text message program providing general mental health support found that a daily, consistent message was appreciated by most participants [34]. Preferences may vary among individuals and during the targeted postpartum behavior change period versus the maintenance phase, making it difficult to identify the perfect length [35].

However, as suggested by some participants, the option of more tailored content could help reduce some of these challenges. The inability to tailor content has also been noted in other mental health [34] and maternally focused [36] SMS text message programs, with a desire for more personalization and interaction expressed by participants. Perhaps in future iterations, the Essential Coaching for Every Mother program could be designed to allow participants to opt in for additional messaging around certain topics, such as breastfeeding or sleep, and opt out of messaging if they feel they are receiving too much information. For instance, a Canadian antenatal text message program called SmartMom found a similar need from participants, resulting in them creating difference streams of messages in which participants could opt in if they wanted more information on, for example, smoking or pregnancy after previous cesarean [34]. This could be an option for future iterations of the Essential Coaching for Every Mother program as well. Furthermore, a preference for interaction has been found in other SMS text message–based mental health programs [37]. While interaction was considered in the initial development of the Essential Coaching for Every Mother program, this was originally decided against to minimize personnel need and to allow for independent operation. Furthermore, challenges exist for scalability as providing direct access to health care providers would require significant funding to create a dedicated position to allow for this type of interaction. However, if interaction is a key component that participants feel is lacking, this may need to be revisited to determine if and how an interactive component could be added to improve the program’s success.

One interesting piece of feedback from participants was that messages were sent in blocks of 3, rather than 1 long message, which was done purposefully. The TextIt platform that was used to program the messages has an upper limit of 160 characters per message, and if a longer message was sent, it may be sent out of order, making it more confusing for participants. Thus, long blocks of content were purposely split into messages under 160 characters to ensure that messages were sent in order, with the goal of improving readability and legibility of health information. A different platform that can send out longer threads of messages could be explored in the future to keep the information in order while minimizing the number of notifications that participants receive when the messages are delivered.

In both the feasibility study [38] and this study, participants requested that the program go on longer than 6 weeks and be available to other care providers, such as their partner. A study by Demirci et al [37] providing SMS text message–based breastfeeding support in the postpartum period also identified that their participants wanted the program to extend beyond 8 weeks and provide similar messaging for partners [39]. The team is currently working on a version for partners as well as an extension beyond 6 weeks. The extension beyond 6 weeks could also address additional content concerns, as there would be more time to offer information without including additional messages in the first 6 weeks. In terms of messages for partners, evidence from SMS4Dads, a postpartum SMS text message program for Australian fathers, shows preliminary effectiveness and positive uptake by fathers [39,40], suggesting that a targeted intervention for Canadian fathers or partners may have similar positive impacts.

Finally, participants also discussed areas where they wanted additional information related to both maternal health (ie, sleep, physical recovery after childbirth, breastmilk pumping, return of menses, and where to go for postpartum support) and newborn care (ie, breastfeeding or pumping, newborn sleep, secure attachment, soothing, infant CPR, and when to seek medical help). The postpartum period has a lot of changes and information needs for women. The Essential Coaching for Every Mother program was originally designed as a 6-week program, requiring a need to balance provision of information and not overwhelming new parents with information. As most participants appreciated the frequency of messages, it would be difficult to add more messages in the base program. However, if an extension of the Essential Coaching for Every Mother program was designed out to 6 months post partum, the addition of these information needs could easily be added.

### Limitations

While this study is able to shed important light on the process evaluation of the RCT, there are limitations. First, owing to unclear variation in reporting between the TextIt program and Twilio program, we were unable to ascertain which messages, if any, were undelivered to participants. Furthermore, owing to the way the SMS text messages are delivered, we were also unable to ascertain if participants read the messages upon delivery. While we anticipate that most participants received and read the text messages, we are unable to confirm that the messages were all read within the designed time frame and as planned. Additionally, there was no fidelity issues reported by the participants (eg, letting us know that they were not receiving the messages and provided feedback on technological challenges faced) and most participants did complete the 6-week follow-up survey, which was primarily sent via SMS text message to participants. This suggests that participants likely received the messages as intended.

Another limitation relates to possible recall bias as participants were asked to provide feedback about the program only after it ended at 6 weeks rather than throughout the program. However, because the program was offered over a short period of time, we anticipate this bias to be limited. We were unable to ascertain why participants withdrew after signing up (ie, they texted “STOP”) and why participants did not complete the baseline survey, which may have provided insight into reasons for study withdrawal.

Finally, we did not collect information on other languages spoken or comprehension of the messages, thus limiting our ability to assess for cultural or language barriers in future iterations of the intervention. Nevertheless, the messages were targeted at an eighth-grade reading level [24], thus increasing the accessibility of the messages to the general population. The sample is also quite heterogenous in terms of race, with 88% of participants identifying as White and only 12% identifying as landed immigrants. While our sample is slightly more diverse than the current demographics of Nova Scotia, where 6% of Nova Scotia’s population identify as a visible minority and 6% as immigrants [41], the findings of the study must be interpreted in this light, with further exploration needed on the impact of the program on non-White, immigrant mothers.

### Conclusions

This process evaluation for the Essential Coaching for Every Mother RCT suggests that the implementation extent and quality were satisfactory. Nearly two-thirds of participants received the full program as it was designed. Participants were generally satisfied and all were willing to recommend it to other new mothers. The information provided by participants on the likes and dislikes of the program along with areas for improvement will be used in future iterations of the Essential Coaching for Every Mother program.

## Appendix

Multimedia Appendix 1 CONSORT-eHEALTH checklist (V 1.6.1).

## Abbreviations

CPR: cardiopulmonary resuscitation
mHealth: mobile health
RCT: randomized controlled trial
